# Postoperative delirium is a risk factor of institutionalization after hip fracture: an observational cohort study

**DOI:** 10.3389/fmed.2023.1165734

**Published:** 2023-08-15

**Authors:** François Labaste, François Delort, Fabrice Ferré, Fanny Bounes, Nicolas Reina, Philippe Valet, Cédric Dray, Vincent Minville

**Affiliations:** ^1^Anesthesiology and Intensive Care Department CHU Toulouse, Toulouse, France; ^2^Institut RESTORE UMR 1301-Inserm 5070-CNRS EFS Univ. P. Sabatier, Toulouse, France; ^3^Orthopedic Surgery Department, CHU Toulouse, Toulouse, France

**Keywords:** orthopedic surgery, hip fracture, delirium, dependence, institutionalization

## Abstract

**Introduction:**

Hip fracture is a common clinical problem in geriatric patients often associated with poor postoperative outcomes. Postoperative delirium (POD) and postoperative neurocognitive disorders (NCDs) are particularly frequent. The consequences of these disorders on postoperative recovery and autonomy are not fully described. The aim of this study was to determine the role of POD and NCDs on the need for institutionalization at 3 months after hip fracture surgery.

**Method:**

A population-based prospective cohort study was conducted on hip fracture patients between March 2016 and March 2018. The baseline interview, which included a Mini-Mental State Examination (MMSE), was conducted in the hospital after admission for hip fracture. NCDs were appreciated by MMSE scoring evolution (difference between preoperative MMSE and MMSE at day 5 >2 points). POD was evaluated using the Confusion Assessment Method. The primary endpoint was the rate of new institutionalization at 3 months. We used a multivariate analysis to assess the risk of new institutionalization.

**Results:**

A total of 63 patients were included. Thirteen patients (20.6%) were newly institutionalized at 3 months. Two factors were significantly associated with the risk of postoperative institutionalization at 3 months: POD (OR = 5.23; 95% CI 1.1–27.04; *p* = 0.04) and IADL evolution (OR = 1.8; 95% CI 1.23–2.74; *p* = 0.003).

**Conclusion:**

Only POD but not NCDs was associated with the risk of dependency and institutionalization after hip fracture surgery. The prevention of POD appears to be essential for improving patient outcomes and optimizing the potential for returning home.

## Introduction

Hip fracture is a common clinical problem in geriatric patients often associated with an increased mortality rate and reduced functions (1). Excess mortality following hip fracture is high (1, 2). However, unfavorable outcomes go beyond mortality. Indeed, older individuals sustaining a hip fracture suffer from long-lasting limitations in mobility, activities of daily living, self-care, participation, and quality of life (1). Hip fracture-related cognitive changes, in one form or another, are a frequent postoperative complication (3–5). Postoperative delirium (POD) and neurocognitive disorders (NCDs) are also known to increase mortality, morbidity, and the risk of rehospitalization, especially in elderly patients (6–9). In survival patients, POD and NCDs lead to a loss of independence and a decline in activities of daily living (10, 11).

Therefore, previously independent living older people have a high risk of new admission to a nursing home during the subsequent months. Between 10 and 20% of hip fracture patients are institutionalized following fracture and surgery (1). Age, gender, physical function, social support, and health perception all have been found to be significantly related to outcome following a hip fracture (1, 6, 12). Prefracture cognitive impairment places patients at greater risk for institutionalization (13, 14). However, few data are available to describe the role of postoperative cognitive changes on the risk of long-term care placement need in these surgical patients (6, 15, 16). Moreover, the frailest elderly patients with preoperative dementia or mild cognitive impairment were not always excluded (16–18). To the best of our knowledge, no study has examined POD and postoperative NCDs together to determine the relative importance of each one in a no frailty and no dementia population.

The aim of this study was to assess possible associations between the occurrence of new institutionalization at 3 months and cognitive changes, POD, or postoperative NCDs in non-frail elderly patients operated for hip fracture.

## Materials and methods

### Study design and ethical considerations

The current study is a subgroup analysis of a prospective monocentric trial (APOCOGNIT, ClinicalTrial.gov NCT02574234). Patients were enrolled in the orthopedic service of the University Hospital of Toulouse (Toulouse, France), where an average of 800 hip fracture patients attend every year. The inclusion was performed from March 2016 to March 2018.

APOCOGNIT was approved by the Clinical Research Ethics Committee of the University Hospital of Toulouse in January 2016, with a decision number of 14 7313 02.

Written informed consent was obtained from all patients who agreed to participate in the APOCOGNIT study.

### Participants

For the current study, among APOCOGNIT, all patients who completed the follow-up at 3 months and who lived at home before the fracture were included. Eligible patients were 75 years old or older and diagnosed during regular working hours with a hip fracture that required surgery. Exclusion criteria were patients with a history of dementia or mild cognitive disorders, patients with a preoperative MMSE (Mini-mental State Evaluation) score of <20 (which ranges from 0 to 30), patients with preoperative diagnosis of delirium and sepsis, patients refusing consent, and patients who did not understand the protocol.

### Anesthesia procedure

All patients were operated within 24 h after admission to the hospital. If the operation was delayed more than 24 h, the patients were excluded from this study. All patients with spinal anesthesia (SA) were enrolled. If necessary, general anesthesia was performed for a second time (failure of SA).

### Data collection

#### Preoperative assessment

Patients underwent a standardized interview conducted by an anesthesiologist. Demographic characteristics, behavioral factors, physical function, and coexisting conditions were assessed. In addition, by reviewing the patient's anesthesia records, the American Society of Anesthesiologists Classification was obtained. The Charlson comorbidity index, a weighted sum of 17 medical conditions, was also calculated (19).

Preoperative cognitive functions were measured using the MMSE. This scale was chosen because it is easy to use, as previously described (20). Functional capacities were assessed using the instrumental activities of daily living (IADL), which ranged from 0 (complete dependence) to 8 (complete independence).

#### Postoperative assessments

Postoperative cognitive assessment began on the next day after surgery and continued until discharge. Patients underwent daily assessment for delirium, which was determined according to the Confusion Assessment Method (CAM) diagnostic algorithm (21). The algorithm consists of four clinical criteria: (1) acute onset and fluctuating course, (2) inattention, (3) disorganized thinking, and (4) altered level of consciousness. To define a patient as having POD, both the first and the second criteria have to be present, as well as either the third or the fourth criteria (3, 21). The Confusion Assessment Method (CAM) was used in the French language and validated among the French elderly population (22).

At day 5 or at discharge if that happened before, a new MMSE scoring was performed.

As previously described (3, 20), postoperative NCDs were appreciated using the MMSE scoring evolution (difference between preoperative MMSE and MMSE at day 5 or at discharge).

After discharge, patients or their families were interviewed (via phone conversation) at 3 months to assess the IADL scale and to assess whether patients are newly institutionalized. Institutionalization was the primary outcome of the study and was defined as new admission to nursing home care within 3 months after hospital admission.

### Statistical analysis

APOCOGNIT was an exploratory study designed to investigate the postoperative inflammatory profile of patients in relation to the occurrence of postoperative delirium. For the APOCOGNIT project, the number of subjects included was 105, with the aim of including at least 40 patients with postoperative delirium. We investigated the data of the subgroup of patients with 3-month follow-up.

For descriptive statistics, the results were expressed as median and 95% CI. The study population was then divided into two groups based on the occurrence of institutionalization at 3 months after hip fracture. After verification of the absence of normality of quantitative data (Anderson–Darling test), patient characteristics were compared using non-parametric tests (Mann–Whitney *U*-test for continuous variables and chi-square exact test for categorical variables).

The association between different covariates and dependent variables (institutionalization) was calculated using a multivariate analysis, setting an initial threshold at a *p*-value of <05. After the exclusion of collinear covariates, stepwise regression (backward elimination) was applied, starting with all the variables initially chosen and then progressively removing non-significant ones.

We used XLSTAT^®^ version 2019 1.1 statistical software (Addinsoft 2020). A *p*-value of <0.05 was considered to be statistically significant.

## Results

**Figure 1:**
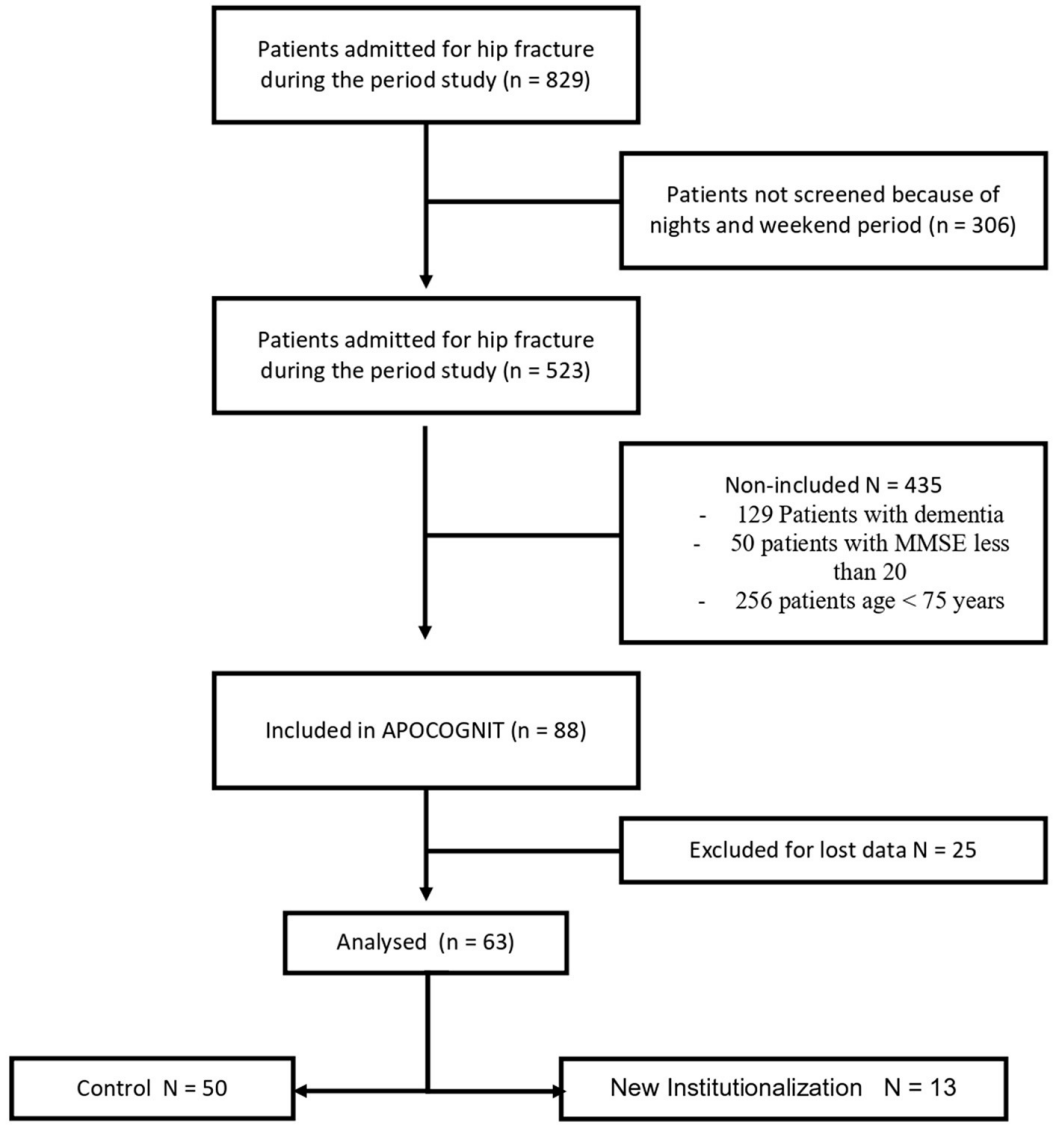
Flowchart diagram of the study.

Among the 88 patients included in APOCOGNIT, 63 patients were included in this study (Figure 1). Seventeen patients were excluded because postoperative cognitive trajectory was not completed and another eight patients were excluded because they were institutionalized before surgery.

Demographic and clinical characteristics of patients are shown in Table 1. The median age of patients was 88 years (IQR 82.5–91), and the majority were women (80.9%). Fourteen patients (22.2%) had a POD, and a decrease in MMSE was observed with a median of 1 (IQR−1–3). The median of length of hospitalization was 9 days (IQR 7–12). Three months after surgery, patients presented a decline in activities of daily living, with a median loss of IADL at 2 (IQR 1–3).

**Table 1:** Demographic and characteristics of patients undergoing hip fracture surgery, *N* = 63.

	**Total**
	***N* = 63**
Age (years)	88.0 (82.5; 91.0)
Sex, women	80.9 (51)
Weight (kg)	62.0 (54.0; 70.0)
IBMI (kg/m^2^)	23.0 (20,5; 25.0)
Preoperative MMSE	25.0 (23.0; 27.0)
Preoperative IADL	6.0 (4.5; 8.0)
Atrial fibrillation	36.5 (23)
Stroke	19.0 (12)
Diabetes	11.1 (7)
Chronic renal failure (MDRD <60 ml/min/1.73 m^2^)	38.0 (19)
High blood pressure	49.2 (31)
Ischemic cardiomyopathy	12.7 (7)
Tumor	15.8 (10)
Hypothyroidism	14.2 (9)
Surgery	58.7 (37)
Chronic cardiac failure	6.3 (4)
Surgery duration, min	90.0 (60.0; 120.0)
General anesthesia	19.0 (12)
ASA score > 2	44.4 (28)
Charlson score	5.0 (4.5; 6.5)
Delirium	22.2 (14)
MMSE at D5 or discharge	24.0 (22.0; 26.0)
MMSE evolution (D5–D1)	1.0 (−1; 3)
Length of hospitalization (days)	9.0 (7; 12)
3 months IADL	3.0 (1; 5)
IADL evolution (3 month–D1)	2.0 (1; 4)

The result is expressed as % (n) or median (Q3; Q1).

BMI, body mass index; MMSE, Mini-mental State Evaluation; IADL, instrumental activities of daily living; MDRD, Modification of Diet in Renal Disease.

Thirteen patients (20.6%) were newly institutionalized 3 months after hip fracture and surgery. The characteristics of patients and the univariate analysis are presented in Table 2.

**Table 2:** New institutionalization at 3 month after hip fracture surgery, *N* = 63.

	**Control**	**New institutionalization**	***p***
	***N*** = **50**	***N*** = **13**	
Age (years)	88.0 (82.2; 91)	88.0 (85; 91)	0.939
Sex, women	80.0 (40.0)	84.6 (11.0)	0.700
Weight (kg)	63.0 (55.0; 70.0)	54.0 (50.0; 68.0)	0.199
IMC (kg/m^2^)	23.5 (21.0; 25.0)	23.0 (20.0; 27.0)	0.772
Preoperative MMSE	25.0 (23.0; 27.5)	23.0 (21.0; 25.0)	0.040^*^
Preoperative IADL	6.0 (4.2; 8.0)	6.0 (5.0; 8.0)	0.985
Atrial fibrillation	34.0 (17)	46.2 (6)	0.422
Stroke	22.0 (11)	7.7 (1)	0.205
Diabetes	11.1 (7)	7.7 (1)	0.647
Chronic renal failure (MDRD <60 ml/min/1.73 m^2^)	38.0 (19)	30.8 (4)	0.626
High blood pressure	50.0 (25)	46.2 (6)	0.805
Ischemic cardiomyopathy	10.0 (5)	23.1 (3)	0.236
Tumor	14.0 (7)	23.1 (7)	0.442
Hypothyroidism	14 (7)	15.4 (2)	0.900
Surgery	56.0 (28)	69.2 (9)	0.382
Chronic cardiac failure	6.3 (4)	0 (0)	0.166
Surgery duration, min	90.0 (60.0; 120.0)	80.0 (60.0; 120.0)	0.772
General anesthesia	20.0 (10)	15.4 (2)	0.700
ASA score > 2	44.0 (20)	46.2 (6)	0.889
Charlson score	5.0 (4.2;7.0)	5.0 (5.0; 6.0)	0.741
Delirium	16 (8)	46.1 (6)	0.028^*^
MMSE at D5 or discharge	24.0 (22.0; 26)	22.0 (20.0; 25.0)	0.038^*^
MMSE evolution (D5–D1)	1 (-1.0; 3.0)	2.0 (−1.0; 3.0)	0.744
Length of hospitalization (days)	8.5 (7.0; 12.0)	9.0 (7.0; 10.0)	0.811
3 months IADL	3.0 (1.0; 5.0)	1.0 (0.0; 2.0)	0.009^*^
IADL evolution (3 month–D1)	2.0 (1.0; 3.0)	5.0 (3.0; 6.0)	0.002^*^

The result is expressed as % (n) or median (Q3; Q1)—*N* = 63.

BMI, body mass index; MMSE, Mini-mental State Evaluation; IADL, instrumental activities of daily living; MDRD, Modification of Diet in Renal Disease.

^*^Data included on multivariate analysis.

There was no association between age (*p* = 0.93), sex (*p* = 0.7), preoperative IADL (*p* = 0.99), or Charlson comorbidity index (*p*= 0.74) and institutionalization. The evolution of MMSE during hospitalization was not different between the two groups (*p* = 0.74).

In multivariate analysis, the best model included POD, preoperative MMSE, and IADL evolution. Cognitive functions evaluated using MMSE at day 5 or at discharge appeared to be not significantly linked to the risk of institutionalization and were not included in our model.

Two factors were significantly associated with the risk of postoperative institutionalization at 3 months: POD (OR = 5.23; 95% CI 1.1–27.04; *p* = 0.04) and IADL evolution (OR = 1.8; 95% CI 1.23–2.74; *p* = 0.003).

Preoperative cognitive functions were not found to be significantly lower in patients with institutionalization at 3 months (OR = 0.78; 95% CI 0.6–1.02; *p* = 0.07). The results are shown in Table 3.

**Table 3:** Multivariate analysis.

	**OR**	**IC 95%**	***p***
Preoperative MMSE	0.79	0.59 – 1.07	0.127
Delirium	5.23	1.01 – 27.04	0.048
IADL evolution (3 month–D1)	1.80	1.23 – 2.74	0.003

MMSE, Mini-mental State Evaluation; IADL, Instrumental activities of daily living.

Hosmer–Lemeshow test = 0.92.

AUC of model = 0.87.

## Discussion

The rate of new institutionalization was 20.6% at 3 months after a hip fracture. Delirium was found to be a risk factor of institutionalization in the multivariate analysis. Both preoperative and postoperative cognitive functions were not associated with the risk to be admitted in a nursing home. Moreover, the decline in activities of daily living was more important in patients who needed institutionalization.

These findings corroborate and extend those of previous investigations of the natural history of hip fracture and its impact on functional recovery (1, 12, 17). Placing the results in the context of earlier studies, the sample was comparable with others in rates of new nursing home placement, and the rates of new institutionalization are between 12 and 27% (23–25).

Moreover, previous studies provided clear evidence that patients recovering from hip fracture experience ongoing limitations in mobility and basic activities of daily living (1). In the present cohort, all patients presented a decline in activities of daily living at 3 months, and not surprisingly, IADL appeared as a predictive factor of the risk of institutionalization.

The current study reported that among patients with postoperative cognitive impairment, only patients who had POD would have a higher institutionalization rate. It has been found that patients who developed POD had poorer recovery within months after the surgery compared with those who did not develop it (26–28). Our study suggests that POD exerts an independent negative influence on functional recovery after hip fracture leading to an increase in poor postoperative outcomes and the need to be admitted to a nursing home. These results were already reported in a meta-analysis including medical and surgical patients, with or without memory impairment before hospitalization (6). Here, we choose to include only emergency surgical patients without preoperative cognitive impairment.

Neither the preoperative cognitive functions nor their evolution during hospitalization appeared to be linked to the risk of institutionalization. Postoperative NCDs and POD were found to be linked in a previous study (15, 20). POD significantly increased the risk of postoperative NCDs, especially in the 1st months. This relationship did not hold in longer term follow-up (29).

Several important clinical findings emerge from our study and have implications for surgeons, anesthetists, geriatricians, and other professionals involved in the care of hip fracture patients. Although early admission to a dedicated orthogeriatric unit seems not to be effective in reducing delirium (30), interdisciplinary orthogeriatric management improve long-term outcome of hip fracture patients (31, 32). In-hospital assessment of delirium seems to be important for identifying patients who are at higher risk of poor outcome after hip fracture surgery. Identification of POD in hip fracture patients can target those in need of more intensive or specialized rehabilitation. Because of their expertise in identifying and treating this condition, the finding provides additional support for geriatrician co-management of these patients (31). A multidisciplinary care bundle, which is shown to reduce the incidence of delirium, has to be set up (33).

Thus, early identification and prevention strategies of POD appear crucial not only to achieve POD prevention but also to improve postoperative outcome and reduce institutionalization. In future, studies on POD treatment and prevention should take these objectives into account.

The current study has several limitations. First, the sample size was small. We were limited in our recruitment because we choose to involve aged patients without dementia, with a high preoperative MMSE. Thus, we selected cooperative patients with a good level of understanding. Second, cognitive evaluation was performed with MMSE, which is not actually recommended (3). The MMSE may lack the necessary sensitivity to identify mild cognitive impairment; thus, it is possible that more sensitive measures would have shown a higher incidence of postoperative NCD. However, MMSE was successfully used previously to study the postoperative cognitive trajectory (20). Finally, the first cognitive evaluation was performed a few hours after fracture. Patients with an acute fracture might find it difficult to concentrate on cognitive tasks because of pain or stress, which would bias toward over-diagnosing cognitive impairment with a preoperative MMSE <20/30. This has led us to exclude more patients than expected.

## Conclusion

In our study, POD and IADL evolution were linked to the risk of institutionalization. POD is a serious and common syndrome in hip fracture patients, and it may markedly affect the outcome and long-term prognosis. Interventions aimed at early identification, prevention, and treatment of this condition seem more necessary than ever. Further research is needed to identify the actual mechanisms by which delirium may contribute to poor outcomes and whether the prevention or reduction of delirium can improve outcomes after hip fracture.

## Data availability statement

The raw data supporting the conclusions of this article will be made available by the authors, without undue reservation.

## Ethics statement

The studies involving human participants were reviewed and approved by University Hospital of Toulouse. The patients/participants provided their written informed consent to participate in this study.

## Author contributions

All authors listed have made a substantial, direct, and intellectual contribution to the work and approved it for publication.
